# The safety and tolerability of cariprazine in long-term treatment of schizophrenia: a post hoc pooled analysis

**DOI:** 10.1186/s12888-017-1459-z

**Published:** 2017-08-24

**Authors:** Henry A. Nasrallah, Willie Earley, Andrew J. Cutler, Yao Wang, Kaifeng Lu, István Laszlovszky, György Németh, Suresh Durgam

**Affiliations:** 1 Saint Louis University, 1438 South Grand Blvd., Suite 105, St. Louis, MO 63104 USA; 2 Allergan, Harborside 5, 185 Hudson Street, Jersey City, NJ 07311 USA; 3 Meridien Research, Inc., 8043 Cooper Creek Boulevard #107, Bradenton, FL 34201 USA; 4 Gedeon Richter, Plc, Gyömrői u. 32, Budapest, H-1103 Hungary

**Keywords:** Cariprazine, Schizophrenia, Long-term safety, Atypical antipsychotic, Post hoc analysis, Open-label

## Abstract

**Background:**

Schizophrenia is a chronic and debilitating neuropsychiatric disorder that often requires long-term pharmacotherapy to manage symptoms and prevent relapse. Cariprazine is a potent dopamine D_3_ and D_2_ receptor partial agonist that is FDA-approved in the US for the treatment of schizophrenia and manic or mixed episodes associated with bipolar I disorder in adults; the recommended dose range is 1.5–6 mg/d.

**Methods:**

To further characterize the long-term safety of cariprazine, data from two 48-week open-label, flexible-dose extension studies were pooled for post hoc analyses. Outcomes were evaluated in the pooled safety population (patients who received ≥1 dose of cariprazine during an open-label extension period); findings were summarized using descriptive statistics for the overall cariprazine group and in modal daily dose groups (1.5–3, 4.5–6, and 9 mg/d).

**Results:**

Of the 679 patients in the overall cariprazine safety population, 40.1% completed the study. The only adverse events (AEs) leading to discontinuation of ≥2% of patients in any dose group were akathisia, worsening of schizophrenia, and psychotic disorder. Treatment-emergent AEs (TEAEs) of akathisia, insomnia, weight increased, and headache were reported in ≥10% of the overall population. Mean prolactin levels decreased in all dose groups (overall, −15.4 ng/mL). Clinically insignificant changes in aminotransferase levels and alkaline phosphatase were observed; no dose-response relationship was observed across groups. Mean total (−5.3 mg/dL), low-density lipoprotein (−3.5 mg/dL), and high-density lipoprotein (−0.8 mg/dL) cholesterol levels decreased; no dose-response relationship was observed for metabolic parameters. Mean change in body weight was 1.58 kg; body weight increase and decrease ≥7% occurred in 27% and 11% of patients, respectively. Mean changes in cardiovascular parameters, including blood pressure and pulse, were generally not considered clinically significant. EPS-related TEAEs that occurred in ≥5% of patients were akathisia, tremor, restlessness, and extrapyramidal disorder.

**Conclusion:**

In these post hoc pooled analyses of data from 2 long-term open-label studies, treatment with cariprazine was generally safe and well tolerated. Results support the safety and tolerability of cariprazine within the FDA-recommended dose range of 1.5–6 mg/d for schizophrenia.

**Clinical trials registration:**

NCT01104792, NCT00839852.

**Electronic supplementary material:**

The online version of this article (doi:10.1186/s12888-017-1459-z) contains supplementary material, which is available to authorized users.

## Background

Schizophrenia is a chronic and debilitating neuropsychiatric disorder with high rates of medical morbidity and mortality [1]. To manage symptoms and prevent relapse, long-term pharmacotherapy is often required [2]. Antipsychotic drugs, which are the foundation of treatment in schizophrenia, differ in many properties and cannot therefore be easily categorized by first- or second-generation distinctions [3]. Even antipsychotics introduced since clozapine (ie, the atypical antipsychotics) do not share a similar class-related pharmacologic profile [4]. Although only small differences in efficacy have been observed among antipsychotic agents in clinical trials, there is variability in individual patient response, as well as in the side effect and tolerability profiles [3]. These factors warrant careful consideration when choosing an antipsychotic treatment.

While first-generation agents are associated with high risk of extrapyramidal symptoms (EPS) and sustained prolactin elevation, second-generation agents have shown a propensity for other important adverse effects including weight gain, metabolic and cardiac abnormalities, somnolence, and sedation [5, 6]. Understanding the side effect profile of an antipsychotic is critical since efficacy can only be viewed within the context of tolerability and the ability of the patient to remain on treatment. In this vein, poor tolerability has been identified as a barrier to medication adherence for patients with schizophrenia [7], and medication nonadherence is associated with considerable consequences including greater risk of relapse, longer time to remission, hospitalization, and attempted suicide [8].

Cariprazine is a potent dopamine D_3_ and D_2_ receptor partial agonist with preferential binding to D_3_ receptors; it is FDA-approved in the United States for the treatment of schizophrenia (1.5–6 mg/d) and manic or mixed episodes associated with bipolar I disorder (3–6 mg/d) in adults. Cariprazine has demonstrated preferential binding to D_3_ receptors in vitro [9], and it has shown high in vivo occupancy of both D_3_ and D_2_ receptors at antipsychotic-effective doses in rats [10] and in clinically active dose ranges in patients with schizophrenia [11]. Other atypical antipsychotics, such as aripiprazole, clozapine, olanzapine, and risperidone, show varying levels of in vitro affinity for D_3_ receptors, but fail to show substantial in vivo D_3_ receptor occupancy in patients at clinically relevant doses [12–14]. Cariprazine also acts as an antagonist at serotonin 5-HT_2B_ receptors and as a partial agonist at 5-HT_1A_ receptors, with lower affinity for 5-HT_2A_, 5-HT_2C_, histamine H_1_, and adrenergic α_1_ receptors and negligible affinity at other receptors [9]. This receptor binding profile may provide potential tolerability benefits since some negative side effects, such as sedation and weight gain, have been associated with affinity at the 5-HT_2C_ and H_1_ receptors [15–17], and some cardiovascular AEs may be associated with affinity at adrenergic α_1_ receptors [18]. Of note, cariprazine has a complex pharmacokinetic profile, with 2 major active metabolites, desmethyl cariprazine and didesmethyl cariprazine, and a long effective half-life for the total active moieties [19].

The short-term safety and efficacy of cariprazine in schizophrenia have been demonstrated in 3 randomized, 6-week, double-blind, placebo-controlled, phase II (RGH-MD-16) and phase III (RGH-MD-04 and RGH-MD-05) clinical trials [20–22]; active comparators to assess assay sensitivity were used in RGH-MD-16 (risperidone) and RGH-MD-04 (aripiprazole). The long-term safety and tolerability of cariprazine has been evaluated in two 48-week, open-label, flexible-dose extension studies (RGH-MD-11 [NCT01104792] and RGH-MD-17 [NCT00839852]) [23, 24]. Study RGH-MD-11 included rollover patients from the short-term lead-in studies (RGH-MD-04 and RGH-MD-05) as well as newly enrolled patients, while study RGH-MD-17 only included rollover patients from RGH-MD-16. To further characterize the long-term safety of cariprazine, data from these extension studies were pooled for post hoc analyses.

## Methods

The protocols of the constituent studies included in these pooled post hoc analyses were approved by an institutional review board or ethics committee and the studies were conducted in compliance with guidelines for good clinical practice; all patients gave informed written consent to participate.

Since the long-term studies included in these analyses were flexibly dosed, this post hoc investigation evaluated safety and tolerability parameters in both the overall pooled population and in patient subgroups based on modal daily dose (the most frequently used dose during the study). Modal daily dose groups allowed us to assess long-term cariprazine exposure and the potential for dose-response relationships in various safety parameters within the FDA-recommended dose range. The modal daily dose groups that were evaluated in these investigations were cariprazine 1.5–3 mg/d, 4.5–6 mg/d, and 9 mg/d.

### Study design

Results from 2 long-term, open-label, flexible-dose studies were pooled for these analyses. In RGH-MD-11, cariprazine was administered in a range of 3–9 mg/d and in RGH-MD-17, the dose range was 1.5–4.5 mg/d. Each study was 53 weeks in duration, consisting of a no-drug screening period of up to 7 days, a 48-week open-label flexible-dose cariprazine treatment period, and a 4-week safety follow-up period. Cariprazine was initiated at 1.5 mg/d on day 1 of both studies. In RGH-MD-11, 1.5-mg dose increases could be made on days 2, 3, and 5, and on day 8, a 3-mg increase could be made to the maximum 9 mg/d dose. In RGH-MD-17, 1.5-mg dose increases could be made on days 2 and 3 to reach the 4.5 mg/d maximum dose. Doses were increased only if response was inadequate and there were no tolerability issues; dosage could be decreased by 1.5-mg/d increments at any time due to tolerability issues.

In both studies, all patients were hospitalized during the first week of open-label treatment; thereafter, patients remained hospitalized for an additional 1 or 2 weeks at the discretion of the investigator or they were discharged and followed-up as outpatients. In the event of clinical deterioration, outpatients could be rehospitalized for safety reasons. For patients who completed a lead-in study, the medical, psychiatric, and medication histories obtained at the first lead-in study visit were used in the extension.

### Inclusion criteria

All patients who participated in the long-term safety studies were 18 to 60 years of age, inclusive, and had a current *Diagnostic and Statistical Manual of Mental Disorders*, Fourth Edition, Text Revision (DSM-IV-TR) [25] diagnosis of schizophrenia for a minimum of 1 year. Participants had normal physical examination, clinical laboratory, vital sign, and electrocardiogram (ECG) results or abnormal results that were not considered clinically significant; female patients of childbearing age were not pregnant or breastfeeding. In RGH-MD-11, patients had either completed 6 weeks of double-blind treatment in a lead-in study (RGH-MD-04 [20] or RGH-MD-05 [22]) or were newly enrolled patients; all patients were required to have a score ≤ 25 on the Positive and Negative Syndrome Scale (PANSS) [26] positive subscale score (PANSS items P1 to P7) and a score ≤ 3 on the Clinical Global Impressions-Severity of Illness (CGI-S) [27]. In RGH-MD-17, eligible patients had completed 6 weeks of double-blind treatment in lead-in study RGH-MD-16 [21] and responded to treatment (CGI-S score ≤ 3 and PANSS total score reduction ≥20% from lead-in study baseline). Patients had a designated caregiver to attend study visits with them or provide written documentation verifying medication compliance.

### Exclusion criteria

Typical exclusion criteria for clinical trials in schizophrenia were applied. Briefly, patients were excluded if they had various psychiatric diagnoses other than schizophrenia (eg, other psychotic disorders, bipolar disorder, cognitive disorders, or alcohol or substance abuse or dependence [with the exception of nicotine or caffeine dependence] within 3 months of the study). Patients with treatment-resistant schizophrenia (no symptomatic response to at least 2 antipsychotic trials of adequate dose and duration), suicide risk (ie, attempt within past 2 years or investigator judged), and a first psychotic episode were also excluded. Additionally, patients were excluded for any concurrent medical condition that may have interfered with the conduct of the study, confounded the interpretation of the study results, or endangered the patient’s well being. Existing issues related to safety and tolerability including clinically significant, uncontrolled adverse events (AEs), EPS that were not adequately controlled by EPS medications, and various ophthalmology assessment criteria were exclusionary. Patients who required concomitant treatment with prohibited medications including psychotropic drugs were excluded; lorazepam (for agitation, irritability, hostility, and restlessness), eszopiclone, zolpidem, zolpidem extended release, chloral hydrate, or zaleplon (for insomnia), and diphenhydramine, benztropine or equivalent, or propranolol (for EPS or akathisia) were allowed.

### Outcome assessments

The long-term safety and tolerability of cariprazine were the primary objectives of the extension studies. Safety parameters included AEs, vital signs, clinical laboratory tests, ECGs, physical examinations, and ophthalmologic examinations. Suicidality was assessed using the Columbia-Suicide Severity Rating Scale (C-SSRS) [28] in RGH-MD-11 and the Suicidality Tracking Scale (STS) [29] in RGH-MD-17. EPS and movement disorders were evaluated by AE reports and rating scale assessments: Barnes Akathisia Rating Scale (BARS), [30] Abnormal Involuntary Movement Scale (AIMS), [31] and Simpson-Angus Scale (SAS) [32].

Pooled PANSS findings summarized changes in the symptoms of schizophrenia. Although no efficacy conclusions can be made based on changes in PANSS because of the lack of a comparator group in this open-label study, it is of note that increased (worse) PANSS scores could indicate a worsening of the clinical condition and could also be evaluated as a safety concern.

### Data analysis

Safety analyses were performed on the safety population, which consisted of patients who received at least one dose of cariprazine during the open-label extension period; findings were summarized using descriptive statistics for the overall cariprazine group and in modal daily dose groups (1.5–3, 4.5–6, and 9 mg/d). Analysis of modal daily dose groups were conducted to evaluate potential dose-response relationships in safety parameters. For patients continuing from a lead-in study, the lead-in study safety baseline was used as the baseline for all analyses of safety parameters; for new patients (RGH-MD-11), the baseline was the last value before the first dose of open-label cariprazine.

AEs were classified by the preferred term. For patients who completed a lead-in study, an AE that started during open-label treatment was considered a treatment-emergent adverse event (TEAE) if it was not present before the first dose of double-blind treatment in the lead-in study or if it increased in intensity after the first dose of open-label treatment. For new patients, an AE that started during open-label treatment was considered a TEAE if it was not present before the first dose of open-label treatment or if it increased in intensity after the first dose of open-label treatment.

In the studies that were pooled, efficacy assessments were collected but not categorized as primary, secondary, or additional. Change from baseline to end of treatment in PANSS total score was summarized using descriptive statistics; no inferential statistics were performed.

## Results

### Patient disposition and demographics

There were 679 patients in the pooled safety population; 586 patients were from RGH-MD-11 and 93 were from RGH-MD-17. Of these patients, 416 had not previously been exposed to cariprazine, having either received placebo or an active comparator in a lead-in study, or enrolling directly into RGH-MD-11 as a new patient. Completion rates were higher in the 1.5–3 and 4.5–6 mg/d modal daily dose groups than in the 9 mg/d modal daily dose group (Table 1). The most frequent reason for discontinuation in any group was withdrawal of consent, which occurred at a similar incidence across doses; discontinuation due to AEs was highest in the 9 mg/d and 1.5–3 mg/d dose groups.

**Table 1:** Patient Disposition (Safety Population)

	Cariprazine 1.5–3 mg/d *n* = 170	Cariprazine 4.5–6 mg/d *n* = 361	Cariprazine 9 mg/d *n* = 148	Cariprazine Overall *N* = 679
Completed, n (%)	70 (41.2)	154 (42.7)	48 (32.4)	272 (40.1)
Reasons for discontinuation, n (%)				
Adverse event	28 (16.5)	29 (8.0)	26 (17.6)	83 (12.2)
Insufficient therapeutic response	1 (0.6)	11 (3.0)	13 (8.8)	25 (3.7)
Protocol violation	13 (7.6)	31 (8.6)	10 (6.8)	54 (8.0)
Withdrawal of consent	44 (25.9)	88 (24.4)	38 (25.7)	170 (25.0)
Lost to follow-up	11 (6.5)	29 (8.0)	10 (6.8)	50 (7.4)
Other reasons	3 (1.8)	19 (5.3)	3 (2.0)	25 (3.7)
Entered safety follow-up, (n)%	121 (71.2)	234 (64.8)	102 (68.9)	457 (67.3)

The demographic and baseline characteristics of the safety population are presented in Table 2.

**Table 2:** Patient Demographic and Baseline Characteristics (Safety Population)

Patient Demographic and Schizophrenia Characteristics	Cariprazine 1.5–3 mg/d *n* = 170	Cariprazine 4.5–6 mg/d *n* = 361	Cariprazine 9 mg/d *n* = 148	Cariprazine Overall *N* = 679
Age, mean (SD), years	40.2 (10.6)	37.9 (10.8)	38.0 (11.2)	38.5 (10.9)
Male, n (%)	123 (72.4)	249 (69.0)	99 (66.9)	471 (69.4)
Race, n (%)				
White	71 (41.8)	174 (48.2)	57 (38.5)	302 (44.5)
Black	64 (37.6)	116 (32.1)	60 (40.5)	240 (35.3)
Asian	17 (10.0)	57 (15.8)	24 (16.2)	98 (14.4)
Other	8 (4.7)	10 (2.8)	6 (4.1)	24 (3.5)
Missing^a^	10 (5.9)	4 (1.1)	1 (0.7)	15 (2.2)
Weight, mean (SD), kg	79.26 (19.15)	77.13 (19.15)	82.69 (22.81)	78.88 (20.09)
Body mass index (BMI), mean (SD), kg/m^2^	26.96 (5.56)	26.32 (5.43)	27.82 (6.26)	26.81 (5.68)
Duration of schizophrenia, mean (SD), years	12.9 (10.0)	11.4 (9.5)	13.5 (9.9)	12.2 (9.8)
Age at onset, mean (SD), years	27.2 (9.4)	26.4 (9.2)	24.4 (8.6)	26.2 (9.1)

^a^ Race data were not collected in patients from Romania in study RGH-MD-11 per local regulations

### Safety and tolerability findings

#### Extent of exposure

Mean (SD) duration of treatment was 188.4 (136.8) days in the overall cariprazine group; treatment duration was longest in 4.5–6 mg/d dose group (201.2 [133.7] days) and similar in the 1.5–3 mg/d (174.1 [147.6] days) and 9 mg/d (173.8 [129.1] days) groups. The overall mean daily dose of cariprazine was 5.42 mg; in the 1.5–3 mg/d, 4.5–6 mg/d, and 9 mg/d dose groups it was 3.08 mg, 5.46 mg, and 7.97 mg, respectively. The overall modal daily dose for patients in the 1.5–3 mg modal daily dose group was 1.5 mg/d for 13 (7.6%) patients and 3 mg/d for 157 (92.4%) patients; the overall modal daily dose for patients in the 4.5–6 mg/d modal daily dose group was 4.5 mg/d for 63 (17.5%) patients and 6 mg/d for 298 (82.5%) patients; the overall modal daily dose was 9 mg/d for all 148 patients in the 9-mg/d group. Patient-years exposure (total amount of time exposed to open-label cariprazine, expressed in years) was 350.3 years.

#### Adverse events

Two deaths occurred during the open-label cariprazine studies; neither event was considered related to treatment. In RGH-MD-17, 1 death from suicide occurred in the cariprazine 4.5–6 mg/d dose group (day 327 of treatment); the patient had no history of suicidality and no trigger event was identified. In RGH-MD-11, 1 death from cardiac hypertrophy of unknown etiology occurred 3 days after the initial screening in a newly enrolled patient who had not received study drug; the patient had normal ECG findings at screening and no history of chest pain.

A summary of TEAEs is presented in Table 3. The only AEs that led to discontinuation of ≥2% of patients in any dose group were akathisia, worsening of schizophrenia, and psychotic disorder. Of patients with TEAEs that resulted in discontinuation, 80.3% had events that resolved; the median time to resolution of TEAEs was 12 days overall (1.5–3 mg/d = 11 days; 4.5–6 mg/d = 14 days; 9 mg/d = 14 days). TEAEs of akathisia, insomnia, headache, and weight increased were each reported in ≥10% of patients overall (Table 3). An inverse dose-response relationship was seen for akathisia, which may be due to patient selection bias since patients who did not tolerate lower doses of cariprazine would be unlikely to up-titrate to a higher dose level. Likewise, patients that developed tolerability concerns at higher doses were down-titrated and remained on lower doses where tolerability was improved. Anxiety and tremor were reported in ≥10% of patients in one of the dose groups that is within the FDA-recommended dose range for schizophrenia (1.5–6 mg/d); schizophrenia was reported in ≥10% of patients in the 9 mg/d dose group (10.8%). Somnolence was reported in 26 (3.8%) patients overall (1.5–3 mg/d = 6 [3.5%] patients; 4.5–6 mg/d = 11 [3.0%]; 9 mg/d = 6 [6.1%]); sedation was reported in 21 (3.1%) patients overall (1.5–3 mg/d = 7 [4.1%] patients; 4.5–6 mg/d = 11 [3.0%]; 9 mg/d = 3 [2.0%]). Overall, AEs were considered mild or moderate in 71.1% of patients (1.5–3 mg/d = 74.1%; 4.5–6 mg/d = 70.1%; 9 mg/d = 70.3%) and related to treatment in 59.6% of patients (1.5–3 mg/d = 113 [66.5%] patients; 4.5–6 mg/d = 204 [56.5%]; 9 mg/d = 88 [59.5%]).

**Table 3:** Treatment-Emergent Adverse Events During Open-Label Treatment (Safety Population)

Summary of Adverse Events	Cariprazine 1.5–3 mg/d *n* = 170 n (%)	Cariprazine 4.5–6 mg/d *n* = 361 n (%)	Cariprazine 9 mg/d *n* = 148 n (%)	Cariprazine Overall *N* = 679 n (%)
Patients with ≥1 TEAE	141 (82.9)	287 (79.5)	127 (85.8)	555 (81.7)
Patients with ≥1 SAE	17 (10.0)	37 (10.2)	28 (18.9)	82 (12.1)
Deaths	0	1 (0.3)	0	1 (0.1)
AEs leading to discontinuations	28 (16.5)	29 (8.0)	27 (18.2)^a^	84 (12.4)
AEs leading to study discontinuation in >2% in any group				
Akathisia	4 (2.4)	1 (0.3)	0	5 (0.7)
Schizophrenia	5 (2.9)	7 (1.9)	9 (6.1)	21 (3.1)
Psychotic disorder	2 (1.2)	3 (0.8)	8 (5.4)	13 (1.9)
Incidence of common TEAEs (≥5% in any group)				
Akathisia	40 (23.5)	45 (12.5)	20 (13.5)	105 (15.5)
Insomnia	22 (12.9)	43 (11.9)	25 (16.9)	90 (13.3)
Headache	25 (14.7)	47 (13.0)	15 (10.1)	87 (12.8)
Weight increased	23 (13.5)	38 (10.5)	10 (6.8)	71 (10.5)
Anxiety	10 (5.9)	36 (10.0)	12 (8.1)	58 (8.5)
Tremor	17 (10.0)	19 (5.3)	11 (7.4)	47 (6.9)
Extrapyramidal disorder	11 (6.5)	22 (6.1)	12 (8.1)	45 (6.6)
Schizophrenia	11 (6.5)	12 (3.3)	16 (10.8)	39 (5.7)
Nausea	12 (7.1)	20 (5.5)	6 (4.1)	38 (5.6)
Restlessness	13 (7.6)	17 (4.7)	8 (5.4)	38 (5.6)
Dyspepsia	9 (5.3)	18 (5.0)	10 (6.8)	37 (5.4)
Nasopharyngitis	10 (5.9)	19 (5.3)	5 (3.4)	34 (5.0)
Blood creatine phosphokinase increased	7 (4.1)	17 (4.7)	9 (6.1)	33 (4.9)
Dizziness	8 (4.7)	18 (5.0)	7 (4.7)	33 (4.9)
Psychotic disorder	4 (2.4)	13 (3.6)	13 (8.8)	30 (4.4)
Constipation	8 (4.7)	18 (5.0)	2 (1.4)	28 (4.1)
Somnolence	6 (3.5)	11 (3.0)	9 (6.1)	26 (3.8)
Dry mouth	5 (2.9)	5 (1.4)	11 (7.4)	21 (3.1)
Back pain	2 (1.2)	18 (5.0)	1 (0.7)	21 (3.1)

^a^One cariprazine 9 mg/d patient had an AE resulting in discontinuation that was previously categorized as withdrawal of consent

The most commonly reported serious AEs (SAEs) were worsening of schizophrenia (30 [4.4%] patients) and psychotic disorder (14 [2.1%] patients). In the 1.5–3 mg/d, 4.5–6 mg/d, and 9 mg/d modal daily dose groups, SAEs of schizophrenia (worsening of symptoms) occurred in 8 (4.7%) patients, 12 (3.3%) patients, and 10 (6.8%) patients, respectively; SAEs of psychotic disorder occurred in 2 (1.2%) patients, 5 (1.4%) patients, and 7 (4.7%) patients, respectively.

#### Extrapyramidal symptoms

Extrapyramidal symptoms were evaluated by AEs and rating scale assessments (Table 4). The EPS-related TEAEs (see footnote in Table 4) that occurred in ≥5% of patients were akathisia, tremor, restlessness, and extrapyramidal disorder. EPS-related TEAEs other than akathisia/restlessness were reported in 135 (19.9%) patients (1.5–3 mg/d = 46 [27.1%] patients; 4.5–6 mg/d = 58 [16.1%]; 9 mg/d = 31 [20.9%]). Tardive dyskinesia was reported in 2 patients (1.5–3 mg/d = 1 [0.1%]; 4.5–6 mg/d = 1 [0.1%]). The majority of patients with any EPS-related AEs had events that were considered mild or moderate in cariprazine overall (97.7%) and in each modal daily dose group (1.5–3 mg/d = 96.0%; 4.5–6 mg/d = 99.0%; 9 mg/d = 98.0%).

**Table 4:** Extrapyramidal Symptoms (Safety Population)

	Cariprazine 1.5–3 mg/d *n* = 170	Cariprazine 4.5–6 mg/d *n* = 361	Cariprazine 9 mg/d *n* = 148	Cariprazine Overall *N* = 679
Discontinuation due to EPS-related TEAEs, n (%)				
Any EPS including akathisia and restlessness	7 (4.1)	4 (1.1)	1 (0.7)	12 (1.8)
Any EPS excluding akathisia and restlessness	3 (1.8)	3 (0.8)	1 (0.7)	7 (1.0)
Incidence of common EPS-related TEAEs (≥5 of patients in any group), n (%)^a^				
Akathisia	40 (23.5)	45 (12.5)	20 (13.5)	105 (15.5)
Tremor	17 (10.0)	19 (5.3)	11 (7.4)	47 (6.9)
Restlessness	13 (7.6)	17 (4.7)	8 (5.4)	38 (5.6)
Extrapyramidal disorder	11 (6.5)	22 (6.1)	12 (8.1)	45 (6.6)
EPS rating scales, mean change (SD)^b^				
AIMS total score	0.1 (1.5)	0.0 (1.5)	0.1 (1.6)	0.0 (1.5)
BARS total score	0.3 (1.7)	−0.1 (0.9)	0.1 (1.1)	0.1 (1.2)
SAS total score	0.4 (3.0)	−0.3 (1.7)	0.1 (1.6)	0.0 (2.1)
Patients with treatment-emergent akathisia or parkinsonism, n/N1 (%)^a,c^				
Akathisia (BARS baseline ≤2 and postbaseline >2)	40/162 (24.7)	55/360 (15.3)	24/148 (16.2)	119/670 (17.8)
Parkinsonism (SAS baseline ≤3 and postbaseline >3)	25/162 (15.4)	30/360 (8.3)	17/148 (11.5)	72/670 (10.7)

The preferred terms for EPS-related AEs are akathisia, bradykinesia, cogwheel rigidity, drooling, dyskinesia, dystonia, extrapyramidal disorder, hypokinesia, masked facies, muscle rigidity, muscle tightness, musculoskeletal stiffness, oculogyric crisis, oromandibular dystonia, parkinsonism, restlessness, salivary hypersecretion, tardive dyskinesia, tongue spasm, tremor, trismus, and torticollis

*AIMS* Abnormal Involuntary Movement Scale, *BARS* Barnes Akathisia Rating Scale, *EPS* Extrapyramidal symptoms, *TEAE* Treatment-emergent adverse event, *SAS* Simpson-Angus Scale

^a^Any time during open-label treatment

^b^Mean change from baseline to end of open-label treatment

^c^N1 is the subset of patients with nonmissing baseline and end-of-study values in the specific baseline category during open-label treatment; n is the number of patients who met the criteria during open-label treatment

Akathisia/restlessness was reported in 133 (19.6%) patients (1.5–3 mg/d = 48 [28.2%] patients; 4.5–6 mg/d = 58 [16.1%]; 9 mg/d = 27 [18.2%]). Most akathisia/restlessness was considered mild or moderate overall (97.8%) and resulted in few study discontinuations overall (6 [0.9%] patients) or in any modal dose group (1.5–3 mg/d = 5 [2.9%] patients; 4.5–6 mg/d = 1 [0.3%]; 9 mg/d = 0). The vast majority of akathisia TEAEs occurred in the first 6 weeks of treatment (1.5–3.0 mg/d = 42 cases; 4.5–6 mg/d = 46 cases; 9 mg/d = 18 cases); from weeks 7 to 48, the total number of first-time akathisia events decreased dramatically (1.5–3.0 mg/d = 6 cases; 4.5–6 mg/d = 12 cases; 9 mg/d = 9 cases). Overall, the median time to resolution of akathisia/restlessness after the last dose of cariprazine was 15 days (1.5–3.0 mg/d = 24 days; 4.5–6 mg/d = 8 days; 9 mg/d = 11 days) and there were no SAEs of akathisia, restlessness, or EPS. Medication was used to manage akathisia/restlessness by the majority of cariprazine patients: 48/89 [53.9%] patients with mild akathisia/restlessness, 28/41 [68.3%] patients with moderate akathisia/restlessness, and 2/3 [66.7%]) patients with severe akathisia/restlessness.

Treatment-emergent akathisia (BARS baseline ≤2 and postbaseline >2) and parkinsonism (SAS baseline ≤3 and postbaseline >3) were reported in a greater percentage of patients in the 1.5–3 mg/d dose group than in the higher dose groups.

#### Clinical laboratory and additional safety parameters

A summary of mean changes from lead-in baseline clinical laboratory values and safety parameters is presented in Table 5.

**Table 5:** Changes in Clinical Laboratory and Safety Parameters (Safety Population)

	Cariprazine 1.5–3 mg/d *n* = 170	Cariprazine 4.5–6 mg/d *n* = 361	Cariprazine 9 mg/d *n* = 148	Cariprazine Overall *N* = 679
Metabolic parameters, mean change (SD)				
Total cholesterol, mg/dL	−2.5 (33.0)	−6.1 (29.1)	−6.4 (33.4)	−5.3 (31.1)
Fasting LDL cholesterol, mg/dL	−1.1 (27.9)	−3.9 (25.1)	−4.9 (28.0)	−3.5 (26.4)
HDL cholesterol, mg/dL	−0.9 (10.9)	−1.0 (11.8)	−0.3 (10.5)	−0.8 (11.3)
Fasting triglycerides, mg/dL	4.4 (114.4)	2.2 (78.5)	−4.4 (74.8)	1.2 (87.2)
Fasting glucose, mg/dL	5.0 (23.6)	3.6 (23.9)	6.3 (20.9)	4.5 (23.2)
Clinically relevant shifts in lipid levels and glucose, n/N1^a^ (%)				
Total cholesterol, normal/borderline (<240 mg/dL) to high (≥240 mg/dL)	6/143 (4.2)	11/328 (3.4)	6/132 (4.5)	23/603 (3.8)
Fasting LDL cholesterol, normal/borderline (<160 mg/dL) to high (≥160 mg/dL)	4/126 (3.2)	5/325 (1.5)	7/133 (5.3)	16/584 (2.7)
HDL cholesterol, normal (≥40 mg/dL) to low (<40 mg/dL)	13/120 (10.8)	45/280 (16.1)	10/107 (9.3)	68/507 (13.4)
Fasting triglycerides, normal/borderline (<200 mg/dL) to high (≥200 mg/dL)	10/119 (8.4)	24/301 (8.0)	13/119 (10.9)	47/539 (8.7)
Fasting glucose, normal (<100 mg/dL) to high (≥126 mg/dL)	3/117 (2.6)	11/270 (4.1)	5/110 (4.5)	19/497 (3.8)
Body weight				
Body weight change, kg, mean (SD)	2.38 (4.96)	1.61 (5.43)	0.64 (5.76)	1.58 (5.42)
≥ 7% increase from baseline, n/N1^a^ (%)	47/161 (29.2)	101/360 (28.1)	34/148 (23.0)	182/669 (27.2)
≥ 7% decrease from baseline, n/N1^a^ (%)	12/161 (7.5)	37/360 (10.3)	24/148 (16.2)	73/669 (10.9)
Clinical laboratory parameters, mean change (SD)				
Prolactin, ng/mL	−13.6 (26.4)	−17.1 (47.6)	−13.3 (29.3)	−15.4 (39.6)
Alanine aminotransferase, U/L	6.0 (44.6)	0.9 (20.8)	2.0 (16.5)	2.4 (27.8)
Aspartate aminotransferase, U/L	1.8 (23.6)	0.1 (12.7)	0.1 (9.4)	0.5 (15.5)
Alkaline phosphatase, U/L	−0.6 (13.2)	−4.4 (32.7)	−1.4 (16.5)	−2.8 (26.1)
Total bilirubin, mg/dL	0.01 (0.27)	0.03 (0.30)	0.02 (0.24)	0.02 (0.28)
Creatine phosphokinase, U/L	−12.5 (218.2)	23.5 (290.6)	40.2 (239.0)	18.5 (264.0)
Cardiovascular parameters, mean change (SD)				
Systolic blood pressure, mmHg	0.6 (11.6)	1.0 (11.1)	0.9 (11.8)	0.9 (11.3)
Diastolic blood pressure, mmHg	0.9 (8.6)	0.0 (8.5)	1.3 (8.9)	0.5 (8.6)
Pulse, bpm	−2.0 (12.4)	−1.7 (12.0)	−2.7 (11.4)	−2.0 (12.0)
Electrocardiogram				
Ventricular heart rate, bpm	−2.0 (14.3)	0.0 (14.7)	−1.7 (13.5)	−0.9 (14.3)
QRS interval, msec	0.9 (6.5)	0.8 (8.2)	−0.8 (7.4)	0.5 (7.7)
PR interval, msec	−2.3 (14.3)	−0.1 (15.1)	−0.4 (15.0)	−0.7 (14.9)
QT interval, msec	4.3 (26.7)	−1.5 (30.5)	0.6 (28.1)	0.4 (29.1)
QTcB interval, msec	−1.3 (22.4)	−1.5 (22.1)	−3.7 (21.0)	−1.9 (21.9)
QTcF interval, msec	0.7 (16.2)	−1.5 (18.0)	−2.2 (17.1)	−1.1 (17.4)
Shift from normotensive to Stage I or Stage II hypertension, n (%)^b^	2/63 (3.2)	3/122 (2.5)	4/46 (8.7)	9/231 (3.9)

*HDL* High-density lipoprotein, *LDL* Low-density lipoprotein, *QTcB* QT interval corrected for heart rate using the Bazett formula, *QTcF* QT interval corrected for heart rate using the Fridericia formula

^a^N1 is the subset of patients who met baseline criteria and had ≥1 nonmissing postbaseline value during open-label treatment; n is the subset of N1 who met the criteria during the open-label treatment period

^b^Normotensive: systolic blood pressure < 120 mmHg and diastolic blood pressure < 80 mmHg; stage I hypertension: systolic blood pressure 140–159 mmHg or diastolic blood pressure 90–99 mmHg; Stage II hypertension: systolic blood pressure ≥ 160 mmHg or diastolic blood pressure ≥ 100 mmHg

Overall, changes in serum chemistry and hematology parameters were not clinically meaningful. Mean prolactin levels decreased in all dose groups. Clinically insignificant increases in alanine aminotransferase (ALT) and aspartate aminotransferase (AST), and clinically insignificant decreases in alkaline phosphatase were observed, with no dose-response relationship seen across groups. Two patients discontinued due to aminotransferase increases (ALT and AST = 1 patient each in the 4.5–6 mg/d and 9 mg/d groups); 1 patient in the 4.5–6 mg/d group discontinued due to an increase in bilirubin. No patient met the criteria for Hy’s Law (ALT or AST ≥3 × upper limit of normal [ULN] concurrent with total bilirubin ≥2 × ULN and alkaline phosphatase <2 × ULN).

Overall, mean creatine phosphokinase (CPK) levels increased during open-label treatment with cariprazine, with the largest increase seen in the 9 mg/d group. Large standard deviations in all groups suggest large fluctuations in CPK levels over time. One patient each in the 1.5–3 mg/d group and the 9 mg/d group discontinued due to clinically significant changes in CPK.

CPK >1000 U/L was noted in 58 of 664 (8.7%) patients; elevations meeting this criterion resolved during the study for 83.1% of patients. CPK >1000 U/L with concurrent positive urine myoglobin was also observed in 5 patients. CPK >5000 U/L was seen in 8 of 664 (1.2%) patients. Observed CPK elevations were not associated with altered renal function or renal failure.

#### Metabolic parameters

Mean total, low-density lipoprotein (LDL), and high-density lipoprotein (HDL) cholesterol levels decreased in all dose groups; no dose-response relationship was observed for metabolic parameters. Clinically relevant shifts from normal/borderline baseline values to high values at the end of treatment for total cholesterol and fasting triglycerides occurred in a similar percentage of patients across dose groups; shifts from normal to high levels in fasting glucose occurred slightly more frequently in the 4.5–6 mg/d and the 6–12 mg/d dose groups than in the 1.5–3 mg/d group.

The percentage of patients with ≥7% increase in body weight was similar in the 1.5–3 mg/d and 4.5–6 mg/d dose groups, and slightly lower for patients in the 9 mg/d group. Of patients who experienced a ≥ 7% increase in body weight, 34% were in normal body mass index (BMI) categories at baseline (BMI >18.5 and <25) or underweight (BMI <18.5); increase in body weight ≥ 7% occurred in 27.6% in the overweight category (BMI 25 to <30) and 16.3% of patients in the obese category (BMI ≥30). Body weight decrease ≥7% was reported with greater frequency in the 9 mg/d dose group than in the 1.5–3 mg/d or 4.5–6 mg/d dose groups.

#### Cardiovascular parameters

Mean changes in cardiovascular parameters, including blood pressure and pulse, were generally not clinically significant. Change in diastolic blood pressure was slightly higher in the 9 mg/d group relative to the lower dose groups, although the increase was not considered clinically significant. Hypertension grouped TEAEs (which included the preferred terms of hypertension and blood pressure increased) occurred in 19 (2.8%) of cariprazine-treated patients overall (1.5–3 mg/d = 6 [3.5%] patients, 4.5–6 mg/d = 7 [1.9%] patients, 9 mg/d = 6 [4.1%] patients). More patients in the 9 mg/d group than in the 1.5–3 mg/d or 4.5–6 mg/d groups shifted from normotensive levels to stage I or II hypertension. Overall, no cardiovascular TEAE was reported in ≥2% of patients and there was no mean increase in QTc interval. A > 60 msec increase in QTcB value occurred in 7 patients (1.0%) overall, while a > 60 msec increase in QTcF value occurred in 2 patients (0.3%) overall. Three patients (0.4%) had a postbaseline QTcB value >500 msec; one of these patients (0.2%) also had a postbaseline QTcF value >500 msec.

Orthostatic hypotension (≥20 mmHg reduction in systolic blood pressure or ≥10 mmHg reduction in diastolic blood pressure while changing from a supine to standing position) was reported in 131/645 (20.3%) of patients overall (1.5–3 mg/d = 33/155 [21.3%] patients; 4.5–6 mg/d = 66/348 [19.0%]; 9 mg/d = 32/142 [22.5%]).

#### Suicidality

For patients in RGH-MD-11, treatment-emergent suicidal ideation based on C-SSRS assessment was noted in 16 (2.8%) cariprazine-treated patients overall (1.5–3 mg/d = 4 [3.0%]; 4.5–6 mg/d = 7 [2.3%]; 9 mg/d = 5 [3.4%]); the majority of C-SSRS suicidal ideation was in the least severe category (“wish to be dead,” no active suicidal thoughts or intent). C-SSRS suicidal behavior was not noted in any cariprazine-treated patient. In RGH-MD-17, no mean change (0.0) in STS scores was observed; among the 7 patients with non-zero STS total scores, no TEAEs related to suicidal ideation or suicidal behavior were reported. TEAEs of suicidal ideation were reported in 4 (0.6%) patients overall (1.5–3 mg/d = 2 [1.2%]; 4.5–6 mg/d = 1 [0.3%]; 9 mg/d = 1 [0.7%]). A completed suicide occurred in a patient in the 4.5–6 mg/d dose group; the event was not considered related to cariprazine.

#### Ophthalmologic parameters

No clinically significant changes were reported on ophthalmologic examination. Blurred vision was the most common ocular AE (1.5–3 mg/d: 2.4%; 4.5–6 mg/d: 1.7%; 9 mg/d: 1.4%). One patient (0.6%) in the 1.5–3 mg/d group had an AE of cataract in the left eye, which was reported on day 337 and considered resolved 38 days after the last dose of cariprazine. Due to its unilateral nature and rapid reversal, it was considered by an independent ophthalmologist to be unrelated to cariprazine treatment. No ocular SAEs were reported.

### Summary of efficacy

Mean (SD) change from the extension study baseline to the end of study in PANSS total score was −4.5 (12.51) for patients in the 1.5–3 mg/d dose group, −7.1 (12.91) for patients in the 4.5–6 mg/d dose group, −2.4 (16.03) for patients in the 9–12 mg/d dose group, and −5.4 (13.70) in the overall cariprazine dose group. Although the primary objective of these open-label studies was to evaluate the long-term safety and tolerability of cariprazine, PANSS total score decreases (improvement) were sustained, suggesting that there was no worsening of schizophrenia symptoms during long-term cariprazine treatment.

## Discussion

Pooled analyses of data from these 2 open-label, 48-week extension studies supported previous findings from studies investigating safety and tolerability of cariprazine in patients with acute exacerbation of schizophrenia. These data may approximate safety findings in the recommended dose range for cariprazine in schizophrenia since almost 80% of patients were in modal daily dose groups in the 1.5–6 mg/d range (1.5–3 mg/d = 170 patients; 4.5–6 mg/d = 361 patients) as opposed to a higher dose (9 mg/d = 148 patients). Approximately 40% of cariprazine-treated patients overall completed the study, with higher completion rates observed in the approved daily dose range (1.5–6 mg/d) than in the 9 mg/d modal daily dose group. The overall completion rate for open-label cariprazine was comparable to rates seen in similarly designed safety studies for other atypical antipsychotics including lurasidone (12-month completion = 36.7% [33]), iloperidone (25-week completion = 41.6% [34], and ziprasidone (12-month completion = 36.9% [35]).

At least 1 TEAE was reported by most patients in the long-term schizophrenia studies; approximately 70% were considered mild or moderate and approximately 60% were considered related to treatment. Discontinuations due to AEs occurred more frequently in the 1.5–3 mg/d and the 9 mg/d dose groups than in the 4.5–6 mg/d group, which is counterintuitive and may have been the result of a patient selection bias. The only AEs that led to discontinuation of ≥2% of patients in any group were akathisia, worsening of schizophrenia, and worsening of psychotic disorder.

Cariprazine has a complex pharmacokinetic profile, with 2 major active metabolites, desmethyl cariprazine and didesmethyl cariprazine. The effective half-life for the total active moieties, which takes into account cariprazine and the 2 major active metabolites, is approximately 1 week [19]. The long half-life triggered initial concern that cariprazine could potentially be associated with dose-related adverse effects and accumulation of the parent drug and active metabolites beyond levels needed for effectiveness. Specifically, post hoc analyses of safety findings from the short-term controlled cariprazine studies in schizophrenia [20–22] showed a dose-response relationship for some TEAEs and clinical laboratory values including akathisia, extrapyramidal disorder, CPK elevations, transaminase elevations, and increases in blood pressure [36]. However, investigating the incidence of safety events in modal daily dose groups helped alleviate concerns about the pharmacokinetics of cariprazine since the risk of these events was determined to be lower at doses ≤6 mg/d than at doses ≥9 mg/d. As such, better tolerability observed at lower cariprazine doses helped establish the FDA-recommended dose range of 1.5–6 mg/d for treating schizophrenia. Results from the post hoc analyses of long-term cariprazine safety data presented here found no new safety concerns related to the pharmacokinetic profile and long half-life of cariprazine. Additionally, there was no evidence of delayed resolution of adverse effects after cariprazine discontinuation. AEs that resulted in discontinuation resolved in 80% of patients, with the median time to resolution of 12 days; the median time to resolution of akathisia/restlessness was 15 days.

The only TEAES reported in ≥10% of patients overall were akathisia, insomnia, headache and weight increased; anxiety and tremor were reported in ≥10% in 1 of the dose groups within the FDA-recommended dose range and worsening of schizophrenia was reported in 11% of patients in the 9 mg/d dose group. The incidence of treatment-related sedation and somnolence, which can interfere with quality of life in a substantial minority of patients with schizophrenia [6], was generally low (3%–4%) and no dose response was observed for these TEAEs. SAEs were nearly twice as likely to occur at the highest dose (9 mg/d) than in the FDA-recommended dose range (1.5–6 mg/d), although the most commonly reported SAEs (worsening of schizophrenia and psychotic disorder) were reported in <5% of patients overall.

Weight gain, dyslipidemia, and glucose dysregulation, which contribute to cardiovascular risk in patients with schizophrenia, are common treatment-related side effects of atypical antipsychotics [37]. Consequently, the effect of cariprazine on these parameters is an important long-term safety consideration. In these post hoc analyses, no dose-response relationship was observed for metabolic parameters and there were no mean increases from baseline in lipid parameters over time with long-term cariprazine treatment. Mean changes from baseline to the end of long-term treatment for fasting glucose (4.5 mg/dL) and the proportion of patients with treatment-emergent significant changes in lipid parameters were similar to changes observed in the 6-week controlled studies [36]; however, the incidence of significant treatment-emergent changes in fasting glucose tended to increase over time with long-term treatment. The overall mean change from baseline in weight at the end of long-term open-label treatment was small (1.58 kg) and only slightly greater than the weight change observed in the 6-week controlled cariprazine studies in schizophrenia (~1 kg) [20–22]. Although weight increases ≥7% were observed in 23%–29% of cariprazine-treated patients across the modal dose groups during long-term treatment, no weight gain event was an SAE or led to study discontinuation. Unlike data from the 6-week controlled trials in schizophrenia, which showed a dose-response relationship for weight gain in cariprazine-treated patients, no consistent pattern of weight-related changes was seen among the dose groups in the pooled 48-week dataset. Weight decreases ≥7% occurred in 11% of cariprazine-treated patients overall, with an apparent dose-response relationship observed (1.5–3 mg/d = 7.5%; 4.5–6 mg/d = 10.3%; 9 mg/d = 16.2%).

Cardiac AEs were minimal among all dose groups, with no individual TEAE occurring in ≥2% of cariprazine-treated patients. There was no mean increase in QTc interval overall; 3 patients had a postbaseline QTcB value >500 msec, one of whom also had a postbaseline QTcF value >500 msec. Shift from normotensive blood pressure to Stage I or Stage II hypertension occurred in a higher percentage of patients in the 9–12 mg/d group than in the lower dose groups.

Akathisia is one of the most commonly recognized treatment-emergent effects associated with antipsychotic treatment, although it is generally reported to be less prevalent with second-generation antipsychotics than with first-generation agents [38–40]. It remains a clinically significant adverse effect however, as shown by results from the Clinical Antipsychotic Trials of Intervention Effectiveness (CATIE) trial, which found that the percentage of patients with schizophrenia who developed akathisia was not significantly different for the intermediate-potency first-generation antipsychotic perphenazine and 4 atypical antipsychotics (olanzapine, quetiapine, risperidone, ziprasidone) [41]. Although the distress of antipsychotic-induced akathisia can be severe enough to adversely affect treatment adherence and long-term patient outcomes [38], it is routinely managed along with other disease-induced or drug-induced symptoms in clinical practice [39].

Akathisia was the most commonly observed TEAE in this pooled 48-week dataset; most occurrences were mild to moderate in severity, considered related to treatment, and rarely led to treatment discontinuation. The first occurrence of akathisia was generally reported within the first 6 weeks of treatment and it resulted in few discontinuations overall (0.9%) or in the modal daily dose groups (0 [9 mg/d group] to 2.9% [1.5–3 mg/d group]). In long-term cariprazine treatment, the majority of patients used medication to manage symptoms of akathisia/restlessness, regardless of the severity of the event. Along with the low rate of discontinuation due to akathisia, this suggests that akathisia was managed by most patients while continuing treatment with cariprazine.

Ocular safety was evaluated in the long-term cariprazine studies in patients with schizophrenia in response to ocular findings from preclinical studies in dogs and rats. In these long-term, open-label pooled analysis, blurred vision was the most commonly reported ocular AE. One patient in the 1.5–3 mg/d group had an AE of cataract that in the judgement of an independent ophthalmologist was not considered a pathological event based on its unilateral nature and rapid reversal, which are atypical of drug-induced cataracts. No ocular SAEs were reported. From these analyses, ophthalmologic AEs associated with cariprazine appear to be rare and not likely related to treatment.

These analyses were subject to the limitations inherent in post hoc analyses, as well as the limitations of open-label study design in which there was no placebo- or active-comparator group. Per the constituent study protocols, safety parameters were not analyzed using inferential statistics. The results of these analyses may have been confounded by a heterogeneous dataset that included patients who had completed a lead-in study as well as patients who were newly enrolled; additionally, patients who continued from a lead-in study may have received either placebo, cariprazine, or aripiprazole. Lack of a randomized study population and the use of modal dose groups may have resulted in patient selection bias. A tolerability bias in favor of patients in the high modal daily dose group may have existed since patients with tolerability issues at lower doses may have been more likely to discontinue the study and less likely to increase their dosage. Conversely, completion rates may have been effected by a bias against patients in the 9 mg/d dose group in reference to discontinuations due to insufficient therapeutic response, as well as discontinuations due to AEs of schizophrenia and psychotic disorder. These events, which occurred at the greatest frequency in the high dose group, may reflect the propensity to increase the dose in patients who do not respond at a lower dose level, indicating potential treatment resistance. Of note, worsening of the underlying condition, in this case schizophrenia and psychotic disorder, is categorized as an AE when in actuality it may be an additional indication of poor treatment response or lack of efficacy. Furthermore, worsening of schizophrenia may be a sign of treatment noncompliance or inconsistency, which is a common problem that may lead to worse outcomes in patients with schizophrenia [8].

## Conclusion

In these post hoc pooled analyses of data from 2 long-term open-label studies, treatment with cariprazine was generally safe and well tolerated. Given the distribution of patients across modal daily dose groups, these results support the safety and tolerability of cariprazine within the FDA-recommended dose range of 1.5–6 mg/d for schizophrenia. Dose-related adverse effects were minimal and no persistent adverse effects after the discontinuation of cariprazine suggest that the complex pharmacokinetics of cariprazine did not appear to affect the long-term safety profile of cariprazine.

## Additional file

Additional file 1: Institutional Review Boards and Independent Ethics Committees. A good clinical practices statement and a table of the Institutional Review Boards (sites in the United States) and Independent Ethics Committees (sites outside of the United States) that approved the protocols. (PDF 166 kb)

## Abbreviations

AE: Adverse event
AIMS: Abnormal Involuntary Movement Scale
ALT: Alanine aminotransferase
AST: Aspartate aminotransferase
BARS: Barnes Akathisia Rating Scale
BMI: Body mass index
CGI-S: Clinical Global Impressions-Severity of Illness
CPK: Creatine phosphokinase
C-SSRS: Columbia-Suicide Severity Rating Scale
DSM-IV-TR: *Diagnostic and Statistical Manual of Mental Disorders*, Fourth Edition, Text Revision
ECG: Electrocardiogram
EPS: Extrapyramidal symptoms
HDL: High-density lipoprotein
LDL: Low-density lipoprotein
PANSS: Positive and Negative Syndrome Scale
QTcB: QT interval corrected for heart rate using the Bazett formula
QTcF: QT interval corrected for heart rate using the Fridericia formula
SAE: Serious adverse event
SAS: Simpson-Angus Scale
STS: Suicidality Tracking Scale
TEAE: Treatment-emergent adverse event
ULN: upper limit of normal
