# Supplementary material for: The safety and tolerability of cariprazine in long-term treatment of schizophrenia: a post hoc pooled analysis
Source: BMC Psychiatry. 2017 Aug 24;17:305. doi: 10.1186/s12888-017-1459-z (PMC5571492; doi:10.1186/s12888-017-1459-z)
Supplement: Additional file 1: — Institutional Review Boards and Independent Ethics Committees. A good clinical practices statement and a table of the Institutional Review Boards (sites in the United States) and Independent Ethics Committees (sites outside of the United States) that approved the protocols. (PDF 166 kb) [file 12888_2017_1459_MOESM1_ESM.pdf]

Additional file 1.

The constituent clinical studies were conducted in full compliance with Food and Drug Administration guidelines for good clinical practice and in accordance with the ethical principles that have their origins in the Declaration of Helsinki. The protocol of each study was approved by an institutional review board at sites in the United States or independent ethics committee at sites outside of the United States (Supplementary table). The studies were conducted in compliance with guidelines for good clinical practice; all patients gave informed written consent to participate.

Table S1. Institutional Review Boards and Independent Ethics Committees

**Institutional Review Board: United States**		
The Copernicus Group IRB	Sharp HealthCare	Western Institutional Review Board
University of Texas Health Science Center at Huston	Human Studies Committee	—
**Independent Ethics Committee: Sites Outside the United States**		
**Colombia**		
Empresa Social del Estado Hospital Mental de Antioquia	C.E.I Campo Abierto Ltda	Sistema Nervioso de Risaralda
**India**		
Shanti Nursing Home	Mental Illness Treatment and Rehabilitation [MITR] Hospital	Poona Hospital and Research Centre
Sheth VS Hospital	Spandana Ethics Committee	VIMHANS Hospital
DMHC Ethics Committee	Kanpur Medical Ethics Committee	SHASHVAT
Jaslok Hospital and Research Centre	Maharasthra Instiute of Mental Health	North Maharashtra Ethics Committee
Andhra Medical College	Nitte University	Jagadguru Shi Shivarathreeshwaru Medical College
Mallikatta Ethical Committee	Lucknow Ethics Committee	Brahmin Mitra Mandal Society
Abhaya Hospital	Mahendru Psychiatric Centre	Kasturba Medical College

Deenanath Mangeshkar Hospital & Research Centre	Sri Venkateshwara Medical College	RK Yadav Memorial and Mental Health De-addiction Hospital
**Malaysia**		
Ministry of Health Malaysia c/o Institute for Health Management		
**Romania**		
Comisia Nationala de Etica Pentru Studiul Clinic al Medicamentului		
**Russia**		
Ministry of Social Health and Development of Russian Federation	St. Petersburg State Healthcare Institution	State Educational Institution of High Professional Education
St. Petersburg Scientific Research Psychoneurological Institute of Roszdrav	Russian Academy of Medical Science Institution Research Centre for Mental Health of RAMS	State Healthcare Institution of city of Moscow ‘Clinical Mental Hospital
Federal Body of Quality Control Drugs	Republican Ethic Committee	Federal Service on Surveillance in Healthcare and Social Development of Russian Federation
**Ukraine**		
The Central Ethic Commission of the Ministry of Health of Ukraine		
